# Integrative Approach of Conventional Physiotherapy, Mulligan's Mobilisation With Movement, and Plyometric Training in a Young Volleyball Athlete After Anterior Cruciate Ligament (ACL) Reconstruction: A Case Report

**DOI:** 10.7759/cureus.54895

**Published:** 2024-02-25

**Authors:** Pratik R Jaiswal, Swapnil U Ramteke, Subrat Samal

**Affiliations:** 1 Sports Physiotherapy, Ravi Nair Physiotherapy College, Datta Meghe Institute of Higher Education & Research, Wardha, IND; 2 Musculoskeletal Physiotherapy, Ravi Nair Physiotherapy College, Datta Meghe Institute of Higher Education & Research, Wardha, IND

**Keywords:** sports physiotherapy, volleyball athlete, mulligan mobilisation with movement, plyometric training, anterior cruciate ligament (acl)

## Abstract

The requirements of volleyball include specialized, strategic, and acrobatic skills. In volleyball, it is thus essential to build maximal power and strength properly. Strengthening has been recommended as an effective means to avoid injuries and build muscle strength. It also enhances one's health in relation to performance in the game. Anterior cruciate ligament (ACL) tears are a common knee injury affecting athletes of all levels. A big problem with injury healing and getting back to sports is that there isn't a tried-and-true protocol or set of steps an athlete should follow following an ACL injury. Plyometric training focuses on the core, hip, and thigh muscles to help with appropriate lower limb alignment and recruiting of muscle. We present a 25-year-old male volleyball athlete who suffered from an ACL tear. This case report emphasises how important sports physiotherapy rehabilitation is for athletes. The case report advances the treatment of ACL injury by a multifaceted approach of Mulligan's mobilisation with movement and plyometric-based interventions.

## Introduction

Volleyball is a challenging sport that requires skill, strategy, and athleticism [1]. During a volleyball match, repeated maximal or nearly maximal jumps, running, diving, dunks, and blocking are common motions [2]. Sports success is influenced by a wide range of complicated factors including sociological, mental, and physical circumstances [3]. In order to play volleyball efficiently, one must achieve their maximal strength and power. Athletes must build their maximal strength in earlier phases of the workout and then properly convert that strength to power as the competition draws near in order to succeed at their peak during matches [4]. In sports, lower limb strength is crucial for improving balance and agility and for producing the power required for explosive actions. Lower body strength is crucial because it promotes quicker and more accurate footwork. The powerful lower body enhances speed when travelling around the court by enabling quick stops and direction changes [5]. This type of exercise focuses on the hip, thigh, core, and abdominal muscles to help with proper lower body alignment and recruiting of muscle patterns [6].

Plyometrics is a training strategy that uses workouts that are explosive and are utilised by athletes in various sports [7]. In plyometric exercises, a muscle is first pre-stretched during an eccentric motion, and then the same muscle and connective tissue are immediately pulled inward during a concentric action. The process is referred to as the "stretch-shortening cycle." It is a type of exercise that combines power with quickness of movement. When a person runs or jumps, their muscles contract in essentially two periods. Muscles undergo two phases: stretching and contraction. The interval between both phases will be shortened by performing these workouts. Substantial energy transfer between the stretch and contraction stages is made possible by a quick cycle time. More force that can be produced by a concentric motion alone is produced by using the elastic energy that has been stored inside the muscle [8]. After the concentric stage, the muscle uses the elastic energies that were saved during the stretch to produce greater effort. Exercises that involve plyometrics may promote central and peripheral neuronal changes that improve joint sense of position and kinaesthetic sensitivity. Eccentric loading may result in the desensitisation of the Golgi tendon organs and sensibility of the spindles of the muscle as a result of a fast stretch and shortening action [9].

The majority of anterior cruciate ligament (ACL) injuries happen when playing sports that involve abrupt stops, changes in path, jumping, or landings. The athlete faces several challenges right away after the injury, including multi-planar biomechanical disparities, missing a season of competition, a protracted and challenging recovery period, and potential performance declines when they return to the sport. Even after rehabilitation is complete, athletes who have had an ACL reconstruction frequently display abnormalities in knee proprioception for the affected limb. Unresolved proprioceptive deficits have been linked to altered lower extremity control and poor postural stability, both of which seem to be risk factors for reinjury [10].

One year after the reconstruction of the ACL, almost two-thirds of the athletes do not regain their preinjury level of performance [11]. For the primary prevention of ACL injuries, neuromuscular training regimens incorporating plyometric, strengthening, and balance training exercises are advised [12]. A common type of joint mobilisation method is Mulligan's mobilisation with movement (MWM), which involves using a manual force to maintain translational or rotational articular glides in order to promote active physiological movement [13].

## Case presentation

A 25-year-old young volleyball player who was a smasher for his team injured his left knee while attempting to land from a jump while playing volleyball. Upon landing, the patient remembers his left leg twisting, hip in external rotation, knee in extension, ankle in dorsiflexion, and feet everted. He also reported hearing a "pop" in his knee at the time of injury. Intense pain and immediate swelling were noted. Further, he was immediately referred to an orthopaedician where on examination, he exhibited intense pain (9 on the visual analogue scale). He was unable to walk and fully flex or extend his knee. Notably, active flexion of the left knee was not possible, and the knee had a limited range of motion (ROM). The ligament stability test could not be performed because of pain. A physical examination and magnetic resonance imaging (MRI) scans revealed a grade 2 ACL tear. The patient elected to undergo surgical reconstruction after which he was referred for sports physiotherapy rehabilitation.

Clinical findings

A specialized examination was done when the patient came to the sports physiotherapy department for rehabilitation. He reported a dull aching pain (5 on the visual analogue scale). On palpation, grade 2 tenderness was present in the left knee. The muscle strength of the affected limb before and after rehabilitation is given in Table 1. The ROM of the affected limb is shown in Table 2. Based on this assessment, an effective treatment plan was designed, combining physical therapy to relieve pain and gain muscular strength. As he was a volleyball player, plyometric training was also incorporated in the later phase.

**Table 1:** Manual muscle testing

		Pre-rehabilitation		Post-rehabilitation	
Joint	Muscles	Left	Right	Left	Right
Hip	Flexor	4	5	5	5
	Extensor	4	5	5	5
	Abductors	4	5	5	5
	Adductors	4	5	5	5
Knee	Flexors	4	5	5	5
	Extensors	4	5	5	5
Ankle	Dorsiflexors	4	5	5	5
	Planterflexors	4	5	5	5

**Table 2:** Range of motion of the left knee

Joint	Movement	Pre-rehabilitation	Post-rehabilitation
Hip	Flexion	0-90°	0-120°
	Extension	0-20°	0-30°
	Abduction	0-25°	0-40°
	Adduction	0-30°	0-30°
Knee	Flexion	5-90°	0-140°
	Extension	90-5°	140-0°
Ankle	Dorsiflexion	0-20°	0-20°
	Plantarflexion	0-40°	0-50°

Radiological investigation

**Figure 1:**
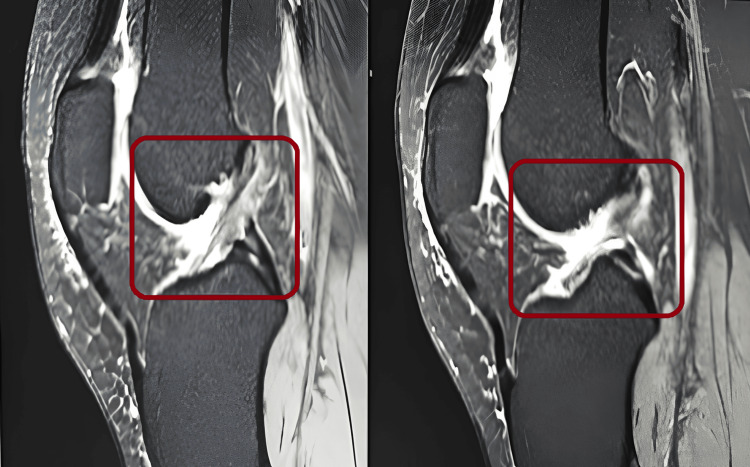
Anterior cruciate ligament tear

MRI was performed for the confirmation of an ACL tear as shown in Figure 1.

Physiotherapy management

A holistic approach to the rehabilitation program (Table 3) and plyometric training (Table 4) was made.

**Table 3:** Physiotherapy rehabilitation ACL: anterior cruciate ligament; ROM: range of motion; R: repetitions; S: sets

Sr. no.	Component affected	Goal	Intervention	Rationale
1	ACL	Facilitate healing and stability	Introduce gentle movements and isometric exercises that target key muscles such as the hamstrings and quadriceps to improve knee mobility	Increases blood flow, decreases edema, and strengthens muscles without putting excessive stress on the healing ligament to improve healing and stability
		Maintain overall joint stability	Isometrics for quadriceps and hamstring (10R × 2S). Dynamic quadriceps (10R × 2S). Twice a day without resistance	Supports the knee joint and increases stability
2	Joint structures	Restoring normal function	Gradual increase in weight bearing	To allow the knee joint to adapt to a gradual increase in load
3	Knee ROM	Limited ROM and stiffness	Mulligan's mobilisation with movement: tibiofemoral posteroanterior glide for knee extension was given (6R × 3S)	Performing knee mobilisation with movement to gain the knee ROM
4	Muscular strength	Regain previous strength	Neuromuscular electrical stimulation: 5 days/week, 50 minutes/day. Symmetrical, biphasic pulses (400 μs at 50 Hz), duty cycle 25%. The patient was asked to perform isometric contractions of the quadriceps	For re-education of quadriceps
5	Patient confidence	Positive attitude	Patient education and encouragement	Educating the patient about the rehab will ultimately benefit him

**Table 4:** Plyometric training S: sets; R: repetitions

Week	Exercise	Dosage
Weeks 1-2	Ankle hops squat jumps (bilateral and unilateral)	3S × 10R
Weeks 3-4	Ankle hops squat jumps (bilateral and unilateral)	4S × 10R
	Jump tuck	2S × 10R
Weeks 5-6	Ankle hops squat jumps (bilateral and unilateral)	5S × 15R
	Jump tuck	3S × 15R
	Hurdle jumps (front, lateral, multi-directional)	3S × 15R
	Lateral high knees with hurdles	3S × 15R
Rest: 30 seconds between sets and two minutes between exercises		

**Figure 2:**
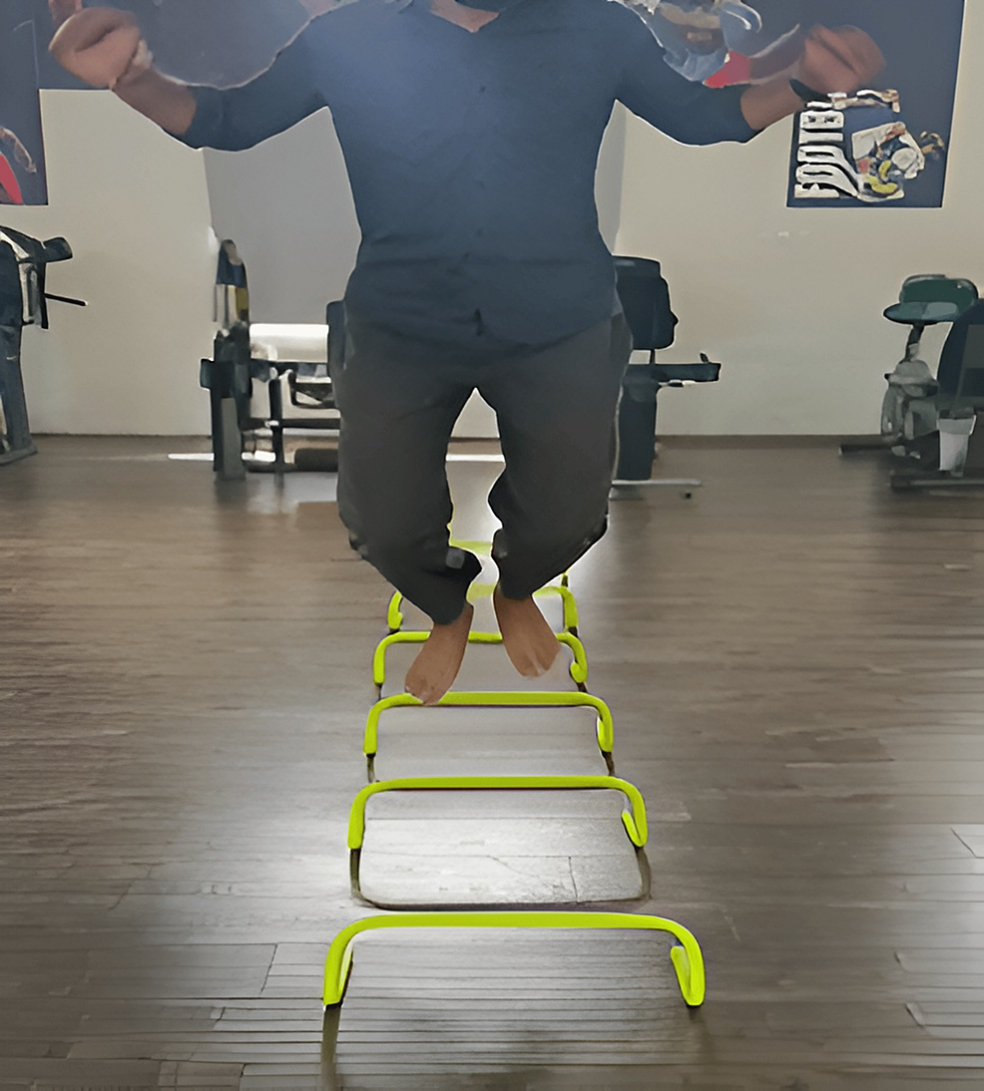
Front barrier jump

**Figure 3:**
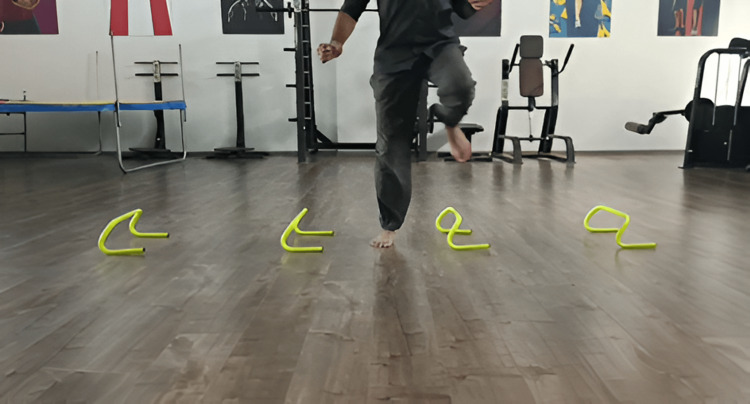
Lateral high knees

**Figure 4:**
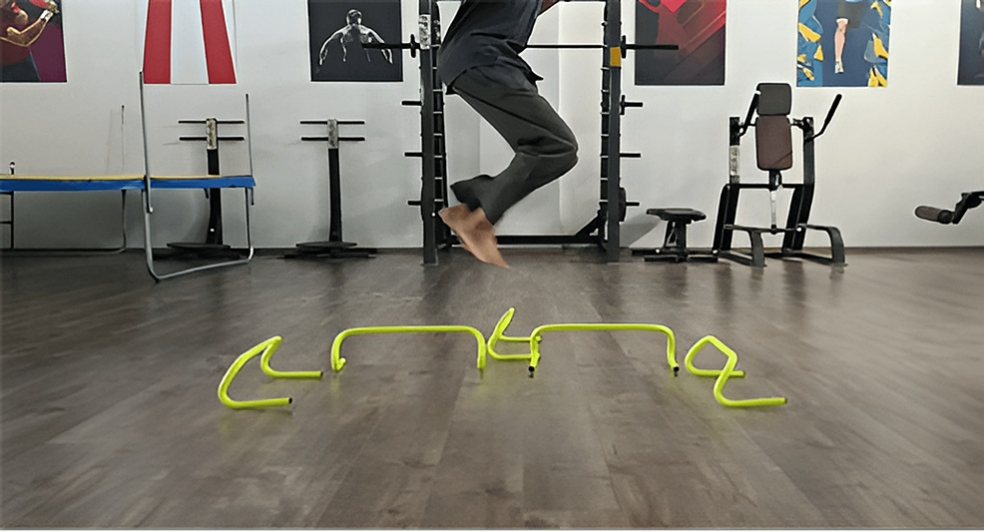
Multi-directional jumps

Figure 2, Figure 3, and Figure 4 show plyometrics.

The pre- and post-training outcome measure scores are shown in Table 5.

**Table 5:** Outcome measures

Scale	Pre-training	Post-training
Vertical jump test	43 cm	61 cm
Y balance test	Right leg: 72%; left leg: 63%	Right leg: 90%; left leg: 88%

## Discussion

In this specific case, combining MWM and plyometric exercises played a key role in accelerating the patient's recovery post-ACL reconstruction. MWM potentially addressed enduring pain and movement dysfunctions that impeded progress with conventional exercises. Incorporating MWM has improved joint mobility and neuromuscular control, paving the way for more efficient movement patterns. Additionally, plyometrics enhanced power and proprioception, further contributing to regaining functional stability and confidence. The combination of MWM and plyometrics, specifically to this patient's needs and challenges, ensured safe and effective rehabilitation progress.

One of the most frequent ligamentous traumas to the knee joint is an ACL injury [14]. Patients who are young and physically active, as well as those who have concurrent disorders and chronic knee instability, ought to undergo surgical therapy [15]. In addition to the kind of surgery, the type and extent of rehabilitation have a major impact on the final result [16]. The early stages of rehabilitation are critical to the result as this is the time when the graft, meniscus, or collateral ligaments begin to restore. Early on in the healing process, it's critical to restore postoperative ROM and muscle strength and initiate neuromuscular training [17]. The majority of sports, including football, karate, handball, badminton, and tennis, benefit from plyometric training [18]. Plyometric exercise is a technique that develops strength and speed to produce more muscular power [19]. Samuel et al. addressed that plyometrics and the application of the stretch-shortening cycle increase the neural and musculotendinous system's ability to produce the most power in a brief period of time, bridging the gap between strength and speed [20]. Lu et al. concluded that male elite badminton players can increase their speed efficiency and dynamic balance by combining balance workouts with plyometrics [21]. Tiong et al. concluded that plyometric exercise may assist badminton players perform better with their overhead clear stroke [19].

## Conclusions

An injured volleyball player showed an excellent recovery after an ACL injury and performed well on the court. The shortcomings in lower limb strength, proprioception, muscular imbalances, and weaknesses from the injury highlighted the complexity of his condition. Enhancing lower limb strength, neuromuscular control, and both static and dynamic stability through plyometric training is a promising way to fortify the ability to deal with volleyball demands while reducing the risk of future injuries. This case emphasises how important customized sports physical therapy programs are for improving athletic performance. Further research is needed to confirm the benefits of combining MWM and plyometrics in ACL rehabilitation. Studies should compare its effectiveness to standard protocols, explore mechanisms of action, define optimal application, and assess safety and feasibility. This could lead to more personalized and effective treatment plans for better patient outcomes. Plyometric training effectively addressed the patient's lower limb strength and power deficits, enhancing his ability to handle the dynamic movements required in volleyball. Neuromuscular control training improved proprioception and stability, reducing the risk of reinjury. The customized program, tailored to the specific demands of volleyball, facilitated a successful return to sport and improved athletic performance.
